# Cullin4 Is Pro-Viral during West Nile Virus Infection of *Culex* Mosquitoes

**DOI:** 10.1371/journal.ppat.1005143

**Published:** 2015-09-01

**Authors:** Prasad N. Paradkar, Jean-Bernard Duchemin, Julio Rodriguez-Andres, Lee Trinidad, Peter J. Walker

**Affiliations:** CSIRO Health and Biosecurity, Australian Animal Health Laboratory, Geelong, Victoria, Australia; The University of Chicago, UNITED STATES

## Abstract

Although mosquitoes serve as vectors of many pathogens of public health importance, their response to viral infection is poorly understood. It also remains to be investigated whether viruses deploy some mechanism to be able to overcome this immune response. Here, we have used an RNA-Seq approach to identify differentially regulated genes in *Culex quinquefasciatus* cells following West Nile virus (WNV) infection, identifying 265 transcripts from various cellular pathways that were either upregulated or downregulated. Ubiquitin-proteasomal pathway genes, comprising 12% of total differentially regulated genes, were selected for further validation by real time RT-qPCR and functional analysis. It was found that treatment of infected cells with proteasomal inhibitor, MG-132, decreased WNV titers, indicating importance of this pathway during infection process. In infection models, the *Culex* ortholog of mammalian Cul4A/B (cullin RING ubiquitin ligase) was found to be upregulated *in vitro* as well as *in vivo*, especially in midguts of mosquitoes. Gene knockdown using dsRNA and overexpression studies indicated that *Culex* Cul4 acts as a pro-viral protein by degradation of *Cx*STAT via ubiquitin-proteasomal pathway. We also show that gene knockdown of *Culex* Cul4 leads to activation of the Jak-STAT pathway in mosquitoes leading to decrease viral replication in the body as well as saliva. Our results suggest a novel mechanism adopted by WNV to overcome mosquito immune response and increase viral replication.

## Author Summary

Mosquitoes are responsible for transmitting a large number of human and livestock viruses, like West Nile, dengue and Japanese encephalitis viruses. Infection of female mosquitoes with these viruses during blood feeding elicits an immune response. It is not known how the viruses manage to replicate in spite of this antiviral response. We used an unbiased transcriptome sequencing approach to identify genes differentially regulated after WNV infection resulting in 265 transcripts from various cellular pathways. Ubiquitin-proteasomal pathway, responsible for protein degradation, was found to be important during viral infection in mosquito cells. Using *in vitro* and *in vivo* infection models, we identified *Culex* Cul4 to be acting as pro-viral protein, increasing viral titers. Knockdown of Cul4 in Culex mosquitoes decreased viral titers in mosquito saliva. Identification of this novel immune evasion mechanism adopted by WNV provides new insights into transmission of arbovirus and interaction of WNV with its mosquito vector.

## Introduction

Flaviviruses, such as West Nile virus (WNV) and dengue virus (DENV), pose a huge burden on public healthcare system worldwide. With more than half of world’s population at risk of infection, the geographic distribution of these mosquito-borne flaviviruses is expanding due to increased travel, trade and climate change [1]. First isolated in Uganda in 1937, WNV is now endemic in parts of Africa, Europe, the Middle East, Asia, Australia and the Americas [2]. Transmitted by *Culex* mosquitoes and causing an acute febrile illness that can lead to severe neurological disease, there is currently no specific vaccine or anti-viral for WNV approved for use in humans [3].

The mammalian response to flavivirus infection has been well studied. Mosquito immune pathways are less well understood but some recent studies have shown that they may play an important role during infection in the vector [4,5]. Although lacking essential components of the mammalian innate and adaptive immune systems, such as interferons, antibodies, B cells, T cells and MHC antigens, mosquitoes have been shown to respond to viral infection by a range of mechanisms including RNA interference (RNAi) and by activation of several evolutionarily conserved signal transduction pathways, include the Toll, Imd/JNK and Jak-STAT [4–7]. Transcriptome analysis using genome-wide microarrays [8–11] have also revealed complex dynamics of mosquito transcripts during infection and identified changes in expression of genes from diverse cellular processes, including ion binding, transport, metabolic processes and peptidase activity. Gene expression is also tissue-specific, with differences reported between midgut and salivary glands [10].

The ubiquitin-proteasomal system is one of the major protein degradation pathways in cells and has been shown to be important during flaviviral infection in mammalian cells [12]. Using a complex set of processes, it affects a myriad of cellular pathways [13]. Ubiquitin itself is a highly conserved 76-amino-acid protein that is highly conserved in sequence from yeast to human [14]. Ubiquitylation is brought about by a cascade of enzymes. E1, ubiquitin activating enzyme, transfers activated ubiquitin to E2, the ubiquitin conjugating enzyme. E3, ubiquitin protein ligase, binds ubiquitin-charged E2 and the substrate, facilitating ligation of ubiquitin to the internal lysine residue on the substrate [15,16]. Substrate specificity is largely determined by the E3 ligase. The E3 family is characterised by the presence of the HECT (Homologous to E6-AP Carboxyl Terminus) [17], RING (Really Interesting New Gene) finger [18], U-box [19] and PHD (Plant Homeo-Domain) [20] or LAP (Leukemia-Associated Protein) finger domains [21]. E3 ligases can work as single or multi-subunit complex. The multi-subunit complexes include a RING-finger subunit and a member of cullin family that binds the RING-finger protein [22]. They also include structural adaptor proteins that link cullin to substrate recognition elements [23].

The ubiquitin-proteasomal pathway has been shown to be important in all stages of viral infection in mammalian cells, including entry, transcription, replication, assembly and exit of virus particles from the cell. A number of ubiquitin enzymes have been found to be involved in the flavivirus infection process. UBE1 has been shown to be important for dengue virus infection in primary human endothelial cells [24]. E3 ubiquitin ligase, CBLL1 (HAKAI) has been found to be important during WNV endocytosis [25], but not in dengue entry [26]. Interestingly, there are also examples of viruses encoding ubiquitin ligases, which lead to evasion of host immunity, by degradation of immune proteins. For example, Kaposi’s sarcoma-associated herpes virus (KSHV) immediate-early transcription factor RTA encodes ubiquitin E3 ligase activity that targets IRF7, a key mediator of type I interferon induction, for proteasome-mediated degradation [27]. As an alternate strategy, viruses also encode adaptors that recruit and redirect host E3 ligases to ubiquitylate host proteins, leading to degradation. Adenoviruses express proteins which recruit a cullin-based E3 ligase to target p53 for degradation [28]. Several paramyxoviruses limit the activity of interferons by targeting STATs for ubiquitylation and degradation via interaction of their highly conserved V proteins and host Cul4A RING E3 ligase [29,30].

Here we use an unbiased approach of transcriptome analysis by deep sequencing of the *Culex* cell transcriptome to identify genes that are differentially regulated following WNV infection. The results show multiple cellular pathways are involved during infection. Using mosquito infection studies *in vitro* and *in vivo*, we determine that the ubiquitin-proteasomal system plays a major role during WNV infection in mosquitoes. We also show that *Culex* cullin (orthologous to mammalian Cul4A/B) is induced by WNV infection and blocks Jak-STAT signalling, increasing WNV replication.

## Results

### Experimental design and analysis of transcriptome sequencing data

Hsu cells (*Culex quinquefasciatus* cell line) were infected with WNV (NY99-4132 strain) at multiplicity of infection (MOI) of 10 and total RNA was collected at 48 h post-infection (hpi) for high-throughput transcriptome sequencing as given in Methods. The experiment was conducted in duplicate and since Pearson correlation coefficients of 0.994 (control) and 0.991 (WNV) indicated close agreement between the biological replicates **(S1 Table)**, data from the replicates was combined for further analysis. The raw sequencing and downstream files were deposited at NCBI with accession code GSE60229.

**Fig 1:**
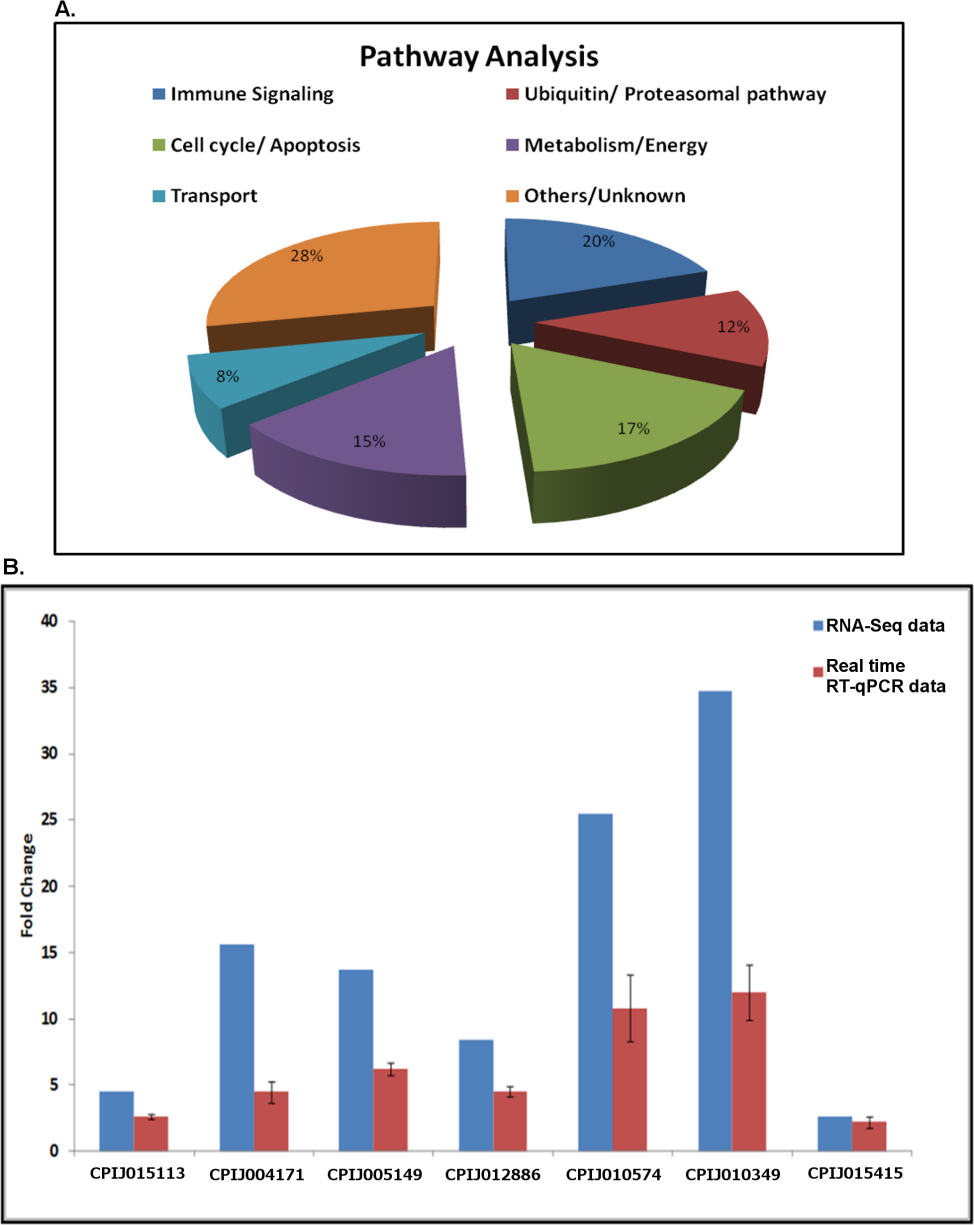
Pathway analysis and validation. **A**. Pathway (molecular and cellular function) analysis was performed for all differentially expressed genes using Ingenuity software. The results are represented here as a pie-chart to indicate the contribution of major cellular pathways during WNV infection. **B**. Hsu cells were infected with WNV and total RNA was collected at 48 hpi. Cells were mock-infected as controls. Real-time RT-qPCR was performed using primers for ubiquitin-proteasomal pathway genes found to be differentially regulated in the RNA-Seq screen. RpL32 (ribosomal protein L32) primers were used as an internal control. The graph was plotted as fold-increase over control (set arbitrarily at 1, not included). Error bars represent standard error from three separate experiments with assays performed in triplicate. The results from the RNA-Seq experiment were also plotted on the graph for comparison.

A total of 265 unique transcripts were identified as differentially accumulated (>2-fold) between control and WNV-infected cells, with 130 transcripts up-regulated and 135 transcripts down-regulated after infection **(S2 Table)**. Ingenuity Pathway Analysis (Ingenuity Systems, www.ingenuity.com) performed on all differentially regulated genes indicated that various pathways and cellular processes were involved during infection, including the immune signalling, the cell-cycle/apoptosis and proteasomal pathways, metabolism genes and the cell transport machinery **(Fig 1A)**. Transcripts, which could not be functionally annotated were grouped as other or unknown. Genes in the ubiquitin-proteasomal pathway comprised 12% of differentially regulated transcripts and were selected for further analysis. To validate the RNA-Seq analysis, the relative abundance of seven differentially up-regulated transcripts in the ubiquitin-proteasomal pathway was determined by real-time RT-qPCR using target-specific primers. The results confirmed that all of the transcripts were up-regulated during infection but the fold-increase was consistently overestimated by RNA-Seq analysis **(Fig 1B)**.

### The proteasomal pathway is required for WNV replication in Culex cells

**Fig 2:**
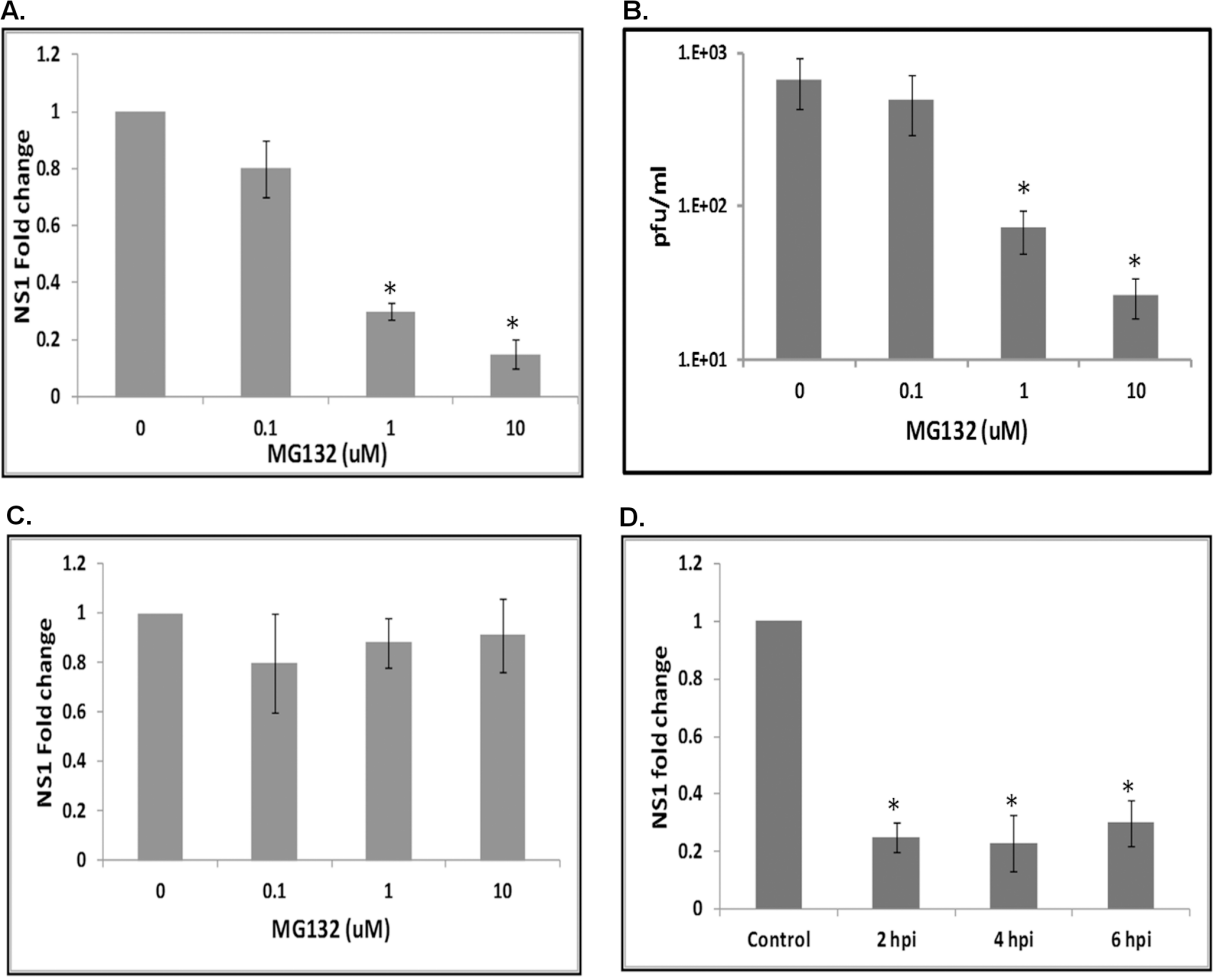
The proteasomal pathway is important during WNV infection. **A**. Hsu cells were pre-treated with MG132 at various concentrations (0.1, 1, 10 μM) for 1 h prior to WNV infection. Total RNA and supernatant medium were collected at 48 hpi. Real-time RT-qPCR was performed using WNV NS1 primers. RpL32 primers were used as an internal control. Error bars represent standard errors from three separate experiments with assays performed in triplicate (Student’s t-test *p < 0.05, comparing between control and MG132 treated cells). **B**. Viral titer estimation by plaque assays conducted on the supernatant media from cells treated as in **A**. Error bars represents standard error from three separate experiments with assays performed in triplicate (Student’s t-test *p < 0.05 compared to control). **C**. Hsu cells were pre-treated with MG132 at various concentrations (0.1, 1, 10 μM) for 1 h prior to infection with WNV at 4°C. Cells were moved to 30°C and total RNA was collected at 6 hpi. Real-time RT-qPCR was performed using WNV NS1 primers. RpL32 primers were used as an internal control. Error bars represent standard error from three separate experiments with assays performed in triplicate (Student’s t-test *p < 0.05, comparing between control and MG132 treated cells). **D**. Hsu cells were infected with WNV, followed by treatment with 10 μM MG132 at 2, 4 or 6 hpi. Total RNA was collected at 48 hpi and real-time RT-qPCR was performed using WNV NS1 primers. RpL32 primers were used as an internal control. Error bars represents standard error from three separate experiments with assays performed in triplicate (Student’s t-test *p < 0.05, comparing between control and MG132-treated cells).

Previous studies have shown that ubiquitin-proteasomal pathway plays an important role in mammalian cells during flavivirus infection [25,31]. Studies have also shown that ubiquitin-related genes are upregulated following bacterial infection in mosquito cells [32]. Experiments were performed to determine the significance of the proteasomal pathway in WNV infected *Culex* cells. Hsu cells were pretreated with 0.1, 1 or 10 μM MG132 (specific proteasomal inhibitor) from 1 h prior to WNV infection (MOI 10), and supernatant media and total RNA from cells were collected at 48 hpi. Real-time RT-qPCR using primers specific for WNV NS1 gene showed a dose-dependent decrease in viral replication, with the NS1 RNA level reducing by more than 80% following treatment with 10 μM MG132 **(Fig 2A)**. Cell viability assay showed no significant toxicity for MG132 at concentrations used in the assay (**S1 Fig**). Plaque assays performed on the supernatant media showed a similar dose-dependent decrease in viral titers, with a 20-fold reduction in cells treated with 10 μM MG132 (30 pfu/ml) compared to untreated controls (600 pfu/ml) **(Fig 2B)**. These results suggest the proteasomal pathway is required for efficient WNV replication in *Culex* cells.

### The proteasomal pathway plays an important role post-viral entry

A previous report suggests the proteasomal pathway has a role during entry of WNV into mammalian cells [25]. To test this in the mosquito system, Hsu cells were pre-treated with MG132 at various concentrations (0.1, 1 and 10 μM), infected with WNV (MOI 10) at 4°C for 30 min and then incubated at 30°C. Real-time RT-qPCR using NS1 primers conducted on total RNA from cells collected at 6 hpi showed no significant effect of MG132 treatment, indicating that the proteasome does not play a role during WNV entry to *Culex* cells **(Fig 2C)**. To determine whether the proteasomal pathway plays significant role post-entry, cells were infected with WNV and treated with 10 μM MG132 from 2, 4, or 6 hpi. Real-time RT-qPCR using NS1 primers conducted on total RNA from cells collected at 48 hpi showed ~80% reduction in viral RNA in all treated samples **(Fig 2D)**. Experiments were also performed to rule out significant contribution by extracellular viral RNA in real time RT-qPCR results (**S2 Fig**). These results suggest involvement of the proteasomal pathway post-viral entry, possibly during viral transcription.

### *Culex* cullin gene is up-regulated following WNV infection of mosquitoes

**Fig 3:**
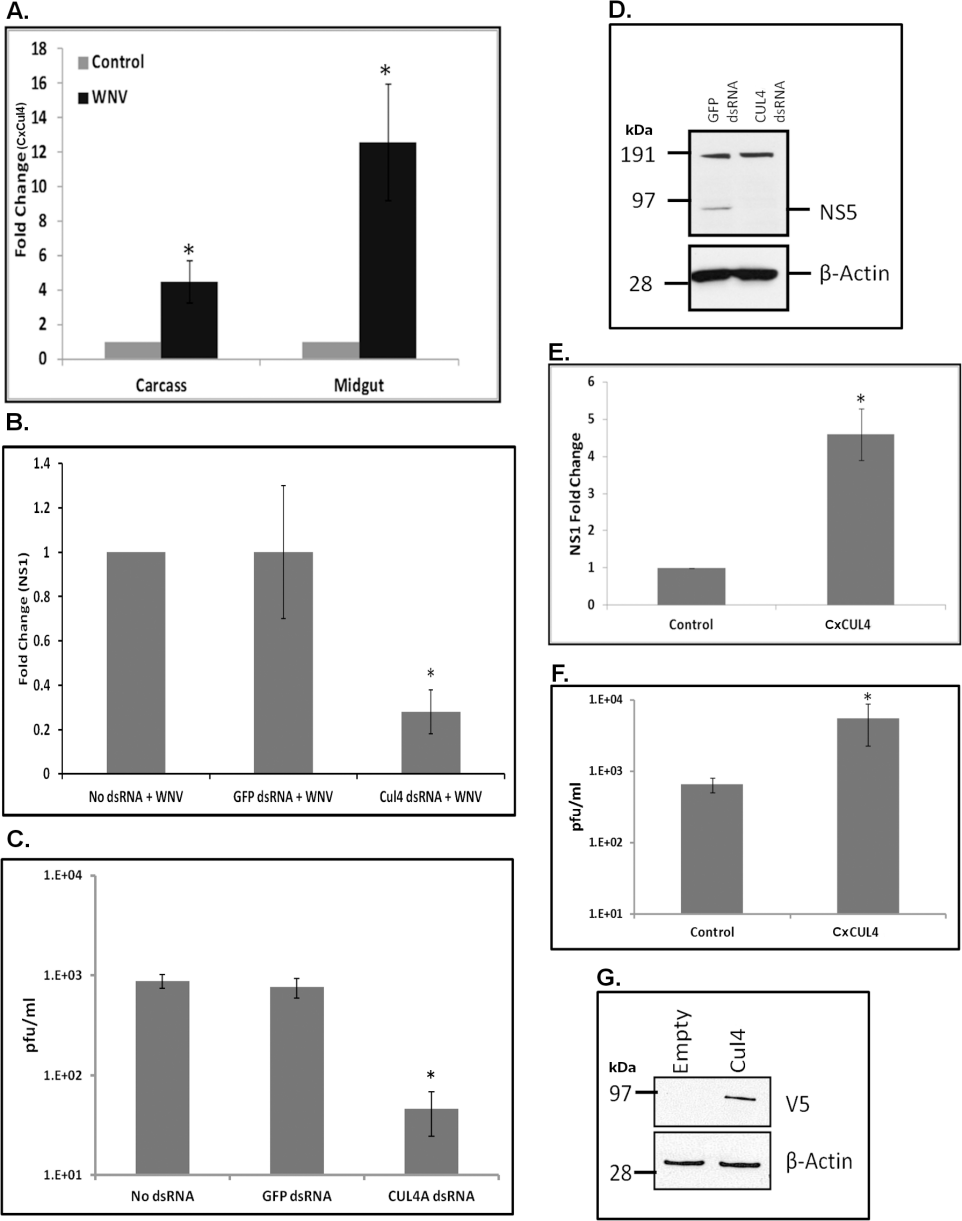
*Culex* cullin (Cul4) is pro-viral. **A**. Female *Culex annulirostris* mosquitoes (n = 40) were infected with WNV by blood feeding. Mosquitoes were micro-dissected at 24 hpi and total RNA was collected from the midgut and the remaining carcass. Real-time RT-qPCR was performed using *Culex* cullin (Cul4) primers. RpL32 primers were used as an internal control. Error bars represent standard error from four pooled mosquitoes with assays performed in triplicate (Student’s t-test *p < 0.05, comparing between control and WNV-infected carcasses and midguts). **B**. Hsu cells were transfected with dsRNA against *Culex* cullin (Cul4 dsRNA). No dsRNA or GFP dsRNA was used as a silencing control. At 24 h post-transfection, the cells were infected with WNV and total RNA was collected 48 hpi. Real-time RT-qPCR was performed using WNV NS1-specific primers. RpL32 primers were used as internal control. Error bars represent standard error from three separate experiments with assays performed in triplicate (*p < 0.05, comparing between No dsRNA, GFP dsRNA and Cul4-knock-down cells). **C**. Viral titer estimation by plaque assays conducted on the supernatant media collected from cells treated as in **B**. **D**. Western blot using anti-WNV NS5 and anti-β-actin antibodies conducted on lysates collected from cells treated as in **B**. Molecular weight markers represented as kDa. The band at around 95 kDa is expected size of WNV-NS5; while band at 191 kDa is non-specific. **E**. Hsu cells were transfected with *Culex* Cul4 overexpression plasmid. Empty vector (Control) was used as a transfection control. At 24 h post-transfection, the cells were infected with WNV and cell lysates were collected at 48 hpi. Total RNA was collected from cells and real-time RT-qPCR was performed using WNV NS1-specific primers. RpL32 primers were used as an internal control. Error bars represent standard error from three separate experiments with assays performed in triplicate (Student’s t-test *p < 0.05, comparing between control and Cul4-transfected cells). **F**. Viral titer estimation by plaque assays conducted on the supernatant media collected from cells treated as in **E**. **G**. Hsu cells were transfected with *Culex* Cul4 overexpression plasmid. Empty vector (Empty) was used as a transfection control. At 24 h post-transfection, the cells were infected with WNV and cell lysates were collected at 48 hpi. Western blot was performed using anti-V5 (detecting *Cx*Cul4 overexpression) and anti-β-actin antibodies. Molecular weight markers represented as kDa.

Amongst the differentially expressed genes in the ubiquitin-proteasomal pathway, CPIJ010574 was highly up-regulated (>20-fold in RNA-Seq and >12-fold in real-time RT-qPCR) following WNV infection in *Culex* cells (**Fig 1B** and **S2 Table**). CPIJ010574 is orthologous to mammalian Cullin4A and Cullin 4B (~70% amino acid identity with both) (**S3 Fig**) and is therefore referred to here as *Cx*Cul4. Cullin-RING ligases, such as Cullin4A, have been found to be upregulated during WNV infection of mammalian cells and implicated in various cellular processes such cell cycle regulation, signal transduction, DNA replication as well as viral replication [33]. Initially, *in vivo* validation of RNA-Seq analysis was performed using a mosquito infection model. Female *Culex annulirostris* mosquitoes were infected with WNV (NY99-4132 strain) by blood-feeding. Total RNA was collected from whole carcasses and dissected midguts at 24 hpi. Real-time RT-qPCR performed using *Cx*Cul4-specific primers showed approximately 4- and 12-fold increases in mRNA in carcass and midgut, respectively, compared with control mosquitoes fed on uninfected blood **(Fig 3A)**.

### *Culex* Cul4 is pro-viral

Gene knock-down experiments were conducted to determine the significance of *Cx*Cul4 during WNV infection. Hsu cells were transfected with long dsRNA against *Cx*Cul4, infected with WNV at 24 h post-transfection, and total RNA and supernatant media were collected at 48 hpi. As a control, cells were either left untransfected (No dsRNA) or were transfected with dsRNA against GFP (GFP dsRNA). Real-time RT-qPCR showed a significant decrease (>80%) in *Cx*Cul4 mRNA, indicating efficient knock-down of the gene (S4 Fig). There was also a significant decrease (>75%) in WNV NS1 RNA in cells treated with *Cx*Cul4 dsRNA, indicating a decrease in viral replication, compared to control (No dsRNA and GFP dsRNA) cells **(Fig 3B)**. Plaque assays conducted on supernatant media showed greater than 10-fold decrease in viral titers following *Cx*Cul4 knock-down (75 pfu/ml) compared with No dsRNA (900 pfu/ml) and GFP dsRNA (800 pfu/ml) **(Fig 3C)**. In a parallel experiment, western blots performed using anti-NS5 antibody showed a significantly lower level of WNV NS5 in cells treated with *Cx*Cul4 dsRNA compared with control cells (GFP dsRNA) **(Fig 3D)**.

To further investigate the role of *Cx*Cul4, Hsu cells were transfected with a plasmid containing *Cx*Cul4 cloned under insect promoter (*OpIE2*), infected with WNV at 24 h post-transfection, and total RNA and supernatant media were collected at 48 hpi. Real-time RT-qPCR showed increased WNV NS1 levels (>4-fold) in cells transfected with *Cx*Cul4 compared with empty vector (Control), indicating increased viral replication **(Fig 3E)**. Plaque assays conducted on supernatant media also showed significantly increased viral titer (> 10-fold) in cells over-expressing *Cx*Cul4 compared with control cells **(Fig 3F)**. In a parallel experiment, western blots using V5 antibody was performed on total cell lysates collected from Hsu cells transfected with *Cx*Cul4 to confirm exogenous expression level of CxCul4 **(Fig 3G)**. Combined, the above data suggest that *Cx*Cul4 plays a significant pro-viral role during WNV infection.

### *Cx*Cul4 blocks Jak-STAT signaling via the proteasomal pathway

**Fig 4:**
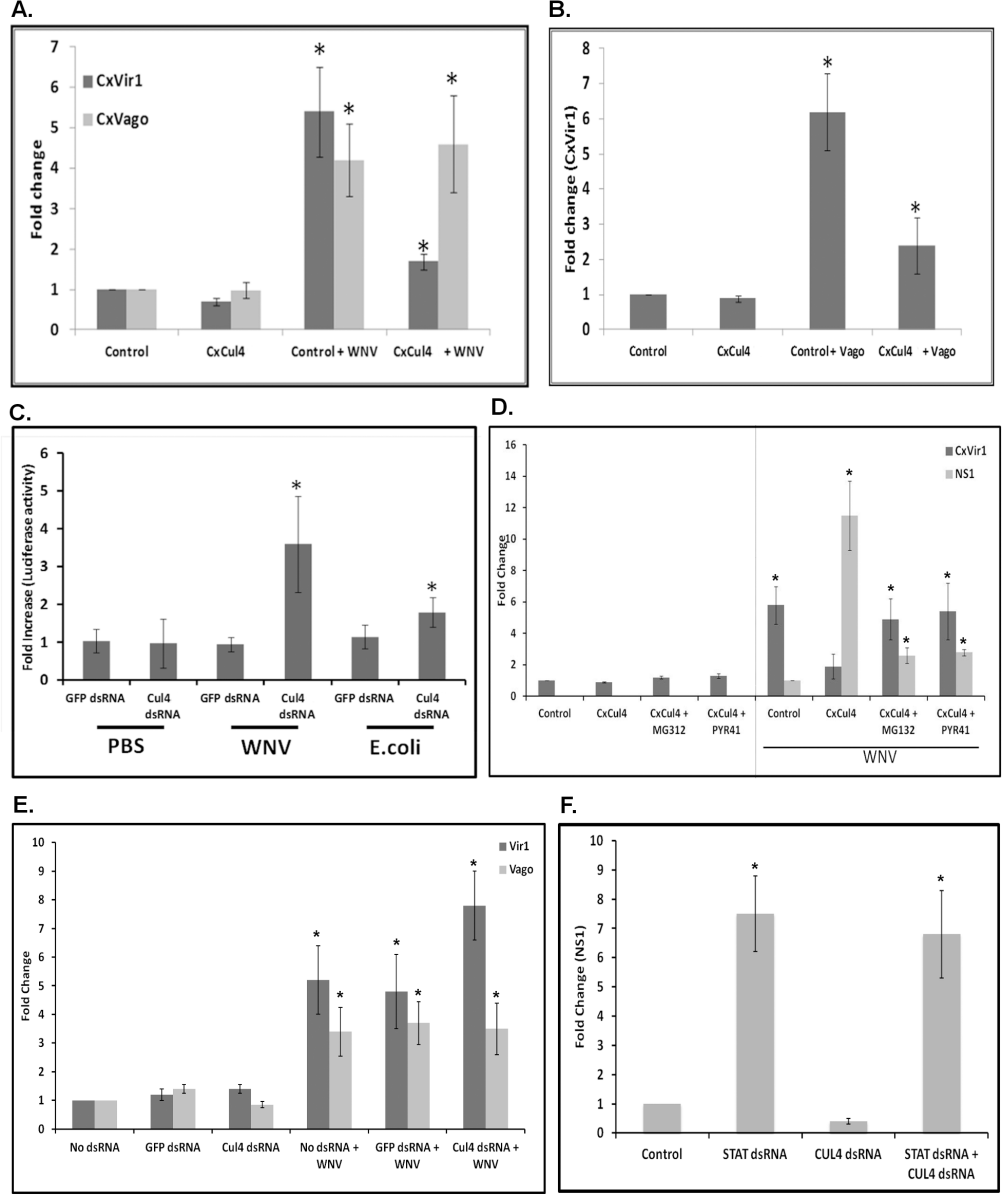
*Culex* cullin (Cul4) blocks the Jak-STAT pathway. **A**. Hsu cells were transfected with plasmid overexpressing *Culex* cullin (*Cx*Cul4). Empty vector (Control) was used as transfection control. At 24 h post-transfection, the cells were infected with WNV and total RNA was collected at 48 hpi. Real-time RT-qPCR was performed using *Culex* Vir1-specific and *Culex* Vago-specific primers. RpL32 primers were used as an internal control. Error bars represent standard error from three separate experiments with assays performed in triplicate (Student’s t-test *p < 0.05, comparing between control and Cul4-overexpressing cells). **B**. Hsu cells were transfected with plasmid overexpressing *Culex* cullin (CxCul4). Empty vector (Control) was used as transfection control. At 24 h post-transfection, the cells were treated with *Cx*Vago-containing medium and total RNA was collected 48 h later. Real-time RT-qPCR was performed using *Culex* Vir1-specific primers. RpL32 primers were used as an internal control. Error bars represent standard error from three separate experiments with assays performed in triplicate (Student’s t-test *p < 0.05, comparing between control and Cul4-overexpressing cells). **C**. Hsu cells were transfected with p6x2DRAF-Luc (STAT reporter) and pAct-Renilla (control) plasmids along with dsRNA against *Culex* Cul4 (Cul4 dsRNA) or GFP (GFP dsRNA). Cells were treated with heat-inactivated *E*. *coli*, WNV or PBS for 1 h. Luciferase activity was measured 16 h post-stimulation. Fold increase over untreated control were plotted after normalising with Renilla transfection control. Error bars represent standard error from six separate samples (Student’s t-test *p < 0.05, comparing between GFP-dsRNA and Cul4-dsRNA cells for each stimulation). **D**. Hsu cells were transfected with plasmid overexpressing *Culex* cullin (CxCul4). Empty vector (Control) was used as a silencing control. At 24 h post-transfection, the cells were infected with WNV and treated with MG132 (1 μM) or PYR-41 (1 μM) or treated with drugs alone. Total RNA was collected at 48 hpi and real time RT-qPCR was performed using *Culex* Vir1 and WNV NS1-specific primers. RpL32 primers were used as internal control. Error bars represent standard error from three separate experiments with assays performed in triplicate (Student’s t-test *p < 0.05, comparing between control and Cul4-overexpressing cells treated with or without inhibitors). **E.** Hsu cells were transfected with dsRNA against *Culex* cullin (Cul4 dsRNA). No dsRNA or GFP dsRNA was used as a silencing control. At 24 h post-transfection, the cells were infected with WNV and total RNA was collected 48 hpi. Real-time RT-qPCR was performed using *Cx*Vir1 (Vir1) and *Cx*Vago (Vago)-specific primers. RpL32 primers were used as internal control. Error bars represent standard error from three separate experiments with assays performed in triplicate (*p < 0.05, comparing between No dsRNA, GFP dsRNA and Cul4-knock-down cells). **F.** Hsu cells were transfected with dsRNA against *Culex* cullin (Cul4 dsRNA) or *Culex* STAT (STAT dsRNA) or both. GFP dsRNA was used as a silencing control (Control). At 24 h post-transfection, the cells were infected with WNV and total RNA was collected 48 hpi. Real-time RT-qPCR was performed using WNV NS1-specific primers. RpL32 primers were used as internal control. Error bars represent standard error from three separate experiments with assays performed in triplicate (Student’s t-test *p < 0.05, comparing between Control and Cul4 dsRNA or STAT dsRNA cells).

Previous reports have shown that mammalian Cul4A plays a role in degradation of STAT2 by an ubiquitin-proteasomal-dependent pathway. To determine whether *Cx*Cul4 plays a similar role during WNV infection, Hsu cells were transfected with plasmid expressing *Cx*Cul4 and infected with WNV 24 h post-transfection. Total RNA was collected 48 hpi and real-time RT-qPCR was performed using primers for *Vir1*, a reporter gene regulated by Jak-STAT pathway [34], and *Cx*Vago, a reporter gene regulated by the TRAF-Rel2 pathway [35]. As expected, expression of both *Cx*Vir1 and *Cx*Vago was upregulated following WNV infection. However, whilst there was no significant difference in *Cx*Vago expression following *Cx*Cul4 overexpression, *Cx*Vir1 upregulation was suppressed significantly in *Cx*Cul4-overexpressing cells compared with empty vector transfected cells **(Fig 4A)**. Previous studies have shown that, once expressed, *Cx*Vago is secreted and activates the Jak-STAT pathway in *Culex* cells [7]. Therefore, Hsu cells were transfected with *Cx*Vago and the supernatant medium was collected 72 h post-transfection. Fresh Hsu cells were then transfected with the *Cx*Cul4 overexpression plasmid and, at 24 h post-transfection, the medium was replaced with medium containing *Cx*Vago to activate the Jak-STAT pathway. Real-time RT-qPCR conducted on total RNA collected 48 h after media replacement showed expression of *Cx*Vir1 was upregulated in control (empty vector transfected) cells but upregulation of *Cx*Vir1 was suppressed significantly in cells overexpressing *Cx*Cul4 **(Fig 4B)**. These results indicate that *Cx*Cul4 functions to inhibit the Jak-STAT pathway in *Culex* cells.

To further confirm that *Cx*Cul4 is a negative regulator of Jak-STAT pathway, Hsu cells were transfected with a plasmid (p6x2DRAF-Luc) containing a Firefly luciferase reporter gene under Drosophila STAT-responsive elements, along with dsRNA against *Cx*Cul4. After 24 h, cells were infected with WNV and luciferase activity was measured 16 hpi. The results showed that *Cx*Cul4 knockdown resulted in increased luciferase activity in WNV-infected cells **(Fig 4C)**. As a positive control, cells were stimulated with heat-inactivated *E*. *coli* for 1 h, which also showed increased luciferase activity.

To determine whether Cul4 acts via the proteasomal pathway, Hsu cells were transfected with the *Cx*Cul4 overexpression plasmid and, at 24 h post-transfection, infected with WNV and treated simultaneously with either MG132 (10 μM) or the ubiquitin activating enzyme E1 inhibitor PYR-41 (10 μM). Real-time RT-qPCR conducted on total RNA collected 48 hpi showed decreased *Cx*Vir1 expression in cells overexpressing *Cx*Cul4 compared with control (empty vector) cells in WNV infected cells. However, treatment of cells overexpressing *Cx*Cul4 with MG132 or PYR-41 resulted in significantly higher levels of *Cx*Vir1 expression compared with untreated cells. Real-time RT-qPCR for WNV NS1 showed there was a decrease in viral replication in cells transfected with *Cx*Cul4 and treated with MG132 or PYR-41 compared with the control (*Cx*Cul4 transfection alone) **(Fig 4D)**. To further determine significance of CxCul4 in STAT signaling, Hsu cells were transfected with dsRNA against *Cx*Cul4. As a control, cells were either not transfected (No dsRNA) or transfected with dsRNA against GFP (GFP dsRNA). Cells were infected with WNV 24 hours post-transfection and total RNA was collected 48 hpi. Real time RT-qPCR using *Cx*Vir1 primers showed increased expression after WNV infection which was further increased in cells transfected with *Cx*Cul4 dsRNA **(Fig 4E)**. *Cx*Vago on the other hand showed increased expression after WNV infection with no further increase in cells with *Cx*Cul4 dsRNA. To determine that action of *Cx*CUl4 is via *Cx*STAT, cells were transfected with *Cx*STAT dsRNA along with dsRNA against *Cx*Cul4. Cells were infected with WNV 24 hours post-transfection. Total RNA was collected 48 hpi and real time RT-qPCR using WNV NS1 primers showed significant increase in cells with *Cx*STAT dsRNA and decrease in cells with *Cx*Cul4 dsRNA. Cells containing both CxCul4 and *Cx*STAT dsRNA showed no decrease in WNV NS1 levels indicating that *Cx*Cul4 action required *Cx*STAT **(Fig 4F)**. These results confirm that pro-viral effect of *Cx*Cul4 is via ubiquitin-proteasomal pathway.

**Fig 5:**
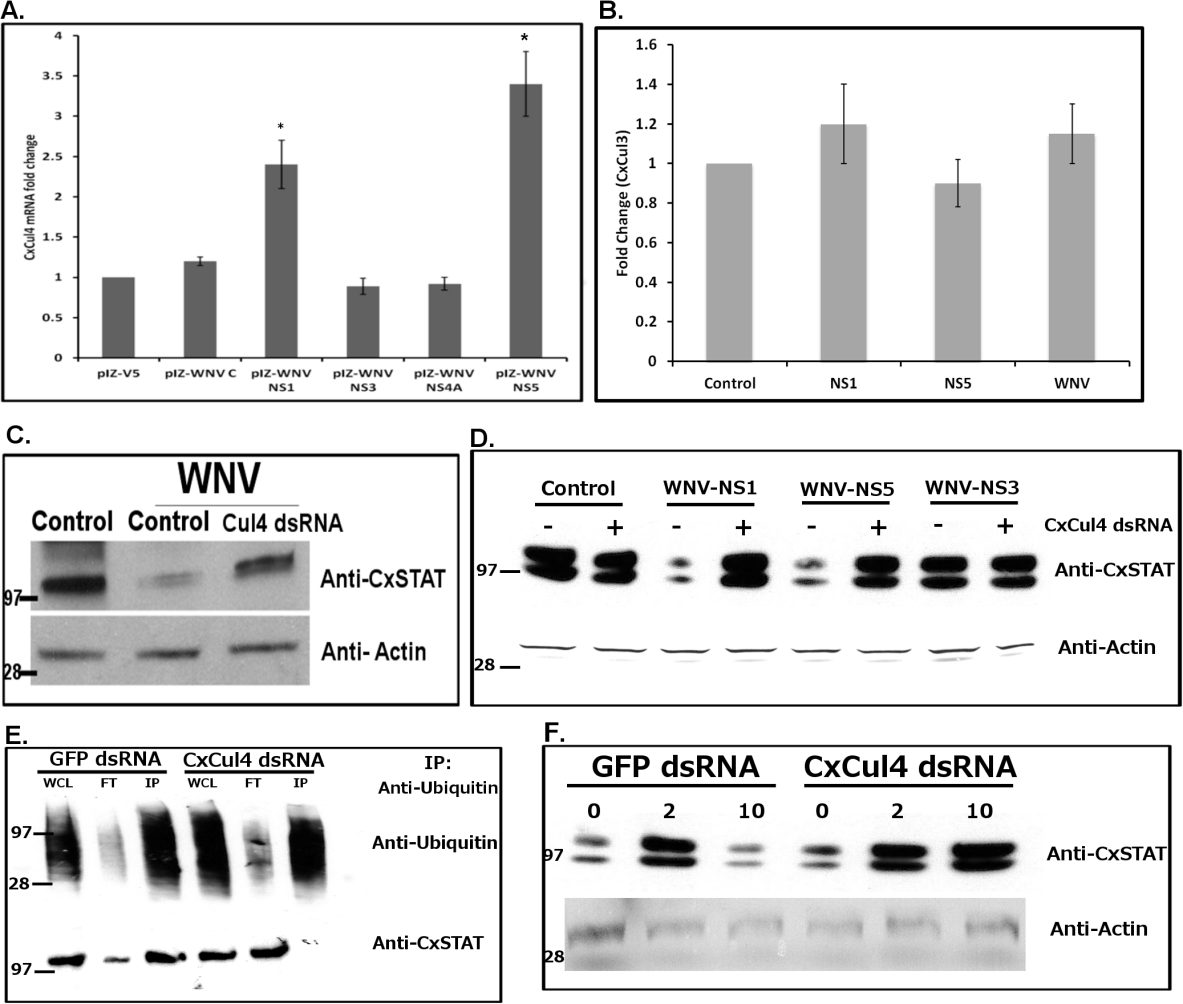
WNV NS1 and NS5 are responsible for *Cx*Cul4 regulation. **A.** Hsu cells were transfected with WNV genes (C-Capsid; NS1, NS3, NS4A or NS5). Total RNA was collected at 24 hours post-transfection and real time RT-qPCR was performed using *Culex* Cul4-specific primers. RpL32 primers were used as internal control. Error bars represent standard error from three separate experiments with assays performed in triplicate (Student’s t-test *p < 0.05, significant from empty vector control). **B.** Real time Rt-qPCr was performed on cells treated as in **A**, using *Culex* Cullin3-specific primers. **C**. Hsu cells were transfected with dsRNA against *Culex* cullin (Cul4 dsRNA). GFP dsRNA was used as a silencing control (Control). At 24 h post-transfection, the cells were infected with WNV and cell lysates were collected 24 hours post-infection. Western blot was performed using anti-*Culex* STAT and anti-beta actin antibodies, with marker representing proteins with known molecular weight (in kDa). The experiment was repeated 3 times and representative blots shown here. **D**. Hsu cells were transfected with WNV genes (NS1, NS5 or NS3) along with dsRNA against *Culex* cullin (+). GFP dsRNA was used as a silencing control (-). Control indicates empty vector transfection. Cell lysates were collected 24 hours post-infection. Western blot was performed using anti-*Culex* STAT and anti-beta actin antibodies. The experiment was repeated and representative blots shown here. **E.** Hsu cells were transfected with dsRNA against *Culex* cullin (Cul4 dsRNA). GFP dsRNA was used as a silencing control (GFP dsRNA). At 24 h post-transfection, the cells were infected with WNV and treated with MG132 (1 μM) and cell lysates were collected 24 hours post-infection. Immunoprecipitation was performed using anti-ubiquitin antibody, followed by Western blot using anti-ubiquitin (Upper) and anti-*Cx*STAT (lower) antibodies. WCL represents whole cells lysate (pre-IP), FT represents flow-through and IP represents immunoprecipitate (eluate). The experiment was repeated twice and representative blots shown here. **F.** Female *Culex annulirostris* mosquitoes were microinjected with dsRNA against Culex Cullin (Cul4). GFP dsRNA was used as a silencing control. At 24 h post-injection, mosquitoes were blood-fed with WNV for 1h. At 2 days and 10 days post-infection, mosquitoes were collected and processed for Western blot using anti-*Cx*STAT and anti-actin antibodies. The experiment was repeated and representative blots shown here.

To determine which WNV protein expression leads to overexpression of CxCul4, Hsu cells were transfected with select individual WNV genes cloned in insect vector (pIZ-V5/His). Total RNA was collected 48 hours post-transfection and real time RT-qPCR was performed using *Cx*Cul4 primers. As a control, real time RT-qPCR was performed using *Cx*RpL32 primers. Western blot was performed on duplicate cells to confirm transfection and protein expression using anti-V5 antibody **(S6 Fig)**. The results showed up-regulation of *Cx*Cul4 expression in cells transfected with WNV-NS1 (2 fold) and WNV-NS5 (3 fold) genes **(Fig 5A)**. This indicates NS1 and NS5 may be involved in upregulation of *Cx*Cul4 after WNV infection in *Culex* cells. As a control, expression of *Cx*Cullin3 (CPIJ011310) was measured after WNV infection or overexpression of WNV NS1 and NS5. Results showed no significant change in expression level **(Fig 5B)**, indicating effect of WNV is specific to *Cx*Cul4.

Experiments were performed to determine whether WNV infection leads to STAT degradation via *Cx*Cul4. Hsu cells were transfected with dsRNA against *Cx*Cul4 and infected with WNV at 24 h post-transfection. Total cell lysates were collected at 24 hours post-infection and Western blot was performed using anti-*Cx*STAT antibody. As a control, cells were either transfected with GFP dsRNA (Control). The results **(Fig 5C)** showed cells infected with WNV showed decreased *Cx*STAT levels, which returned to baseline in cells transfected with dsRNA against *Cx*Cul4. Although, we cannot rule out the possibility that reduced degradation of *Cx*STAT upon *Cx*Cul4 knockdown is due to lower virus replication, these results combined with other data indicate that WNV infection leads to degradation of *Cx*STAT via *Cx*Cul4.

To further establish this, Hsu cells were transfected with WNV-NS1, WNV-NS5 or WNV-NS3 genes with or without transfection with dsRNA against *Cx*Cul4. As a control cells were transfected with dsRNA against GFP. Cell lysates were collected 48 hours post-transfection. Western blot was performed using anti-*Cx*STAT antibody. The results showed decreased STAT levels in cells transfected with WNV-NS1 or WNV-NS5 **(Fig 5D)**. The levels returned to baseline in cells also transfected with dsRNA against *Cx*Cul4. In WNV-NS3 transfected cells, there was no significant change in STAT level. These results indicate that WNV infection of *Culex* cells leads to degradation of *Cx*STAT via *Cx*Cul4, activated by WNV NS1 and NS5.

*Cx*Cul4, ortholog of mammalian Cullin4A/B, has previously been shown to be responsible for degradation of STAT via ubiquitin-proteasomal pathway. Cullin-RING ubiquitin ligases are responsible for ubiquitylation of target proteins. To determine whether degradation of *Cx*STAT via *Cx*Cul4 occurs by ubiquitylation, *Culex* cells were transfected with dsRNA against *Cx*Cul4 (or GFP dsRNA as control). The cells were infected with WNV and cell lysates collected 48 hours post-infection. Immunoprecipitation was performed using anti-ubiquitin antibody, followed by Western blot using anti-ubiquitin and anti-*Cx*STAT antibodies. The results **(Fig 5E)** showed that STAT was co-immunoprecipitated with ubiquitin after WNV infection in control (GFP dsRNA) cells, indicating ubiquitylation of *Cx*STAT. *Cx*STAT was not co-immunoprecipitated with ubiquitin in cells transfected with dsRNA against *Cx*Cul4. The results suggest that *Cx*Cul4 plays a major role in ubiquitylation of *Cx*STAT after WNV infection.

### *Cx*Cul4 knockdown reduces viral titers in mosquito saliva

**Fig 6:**
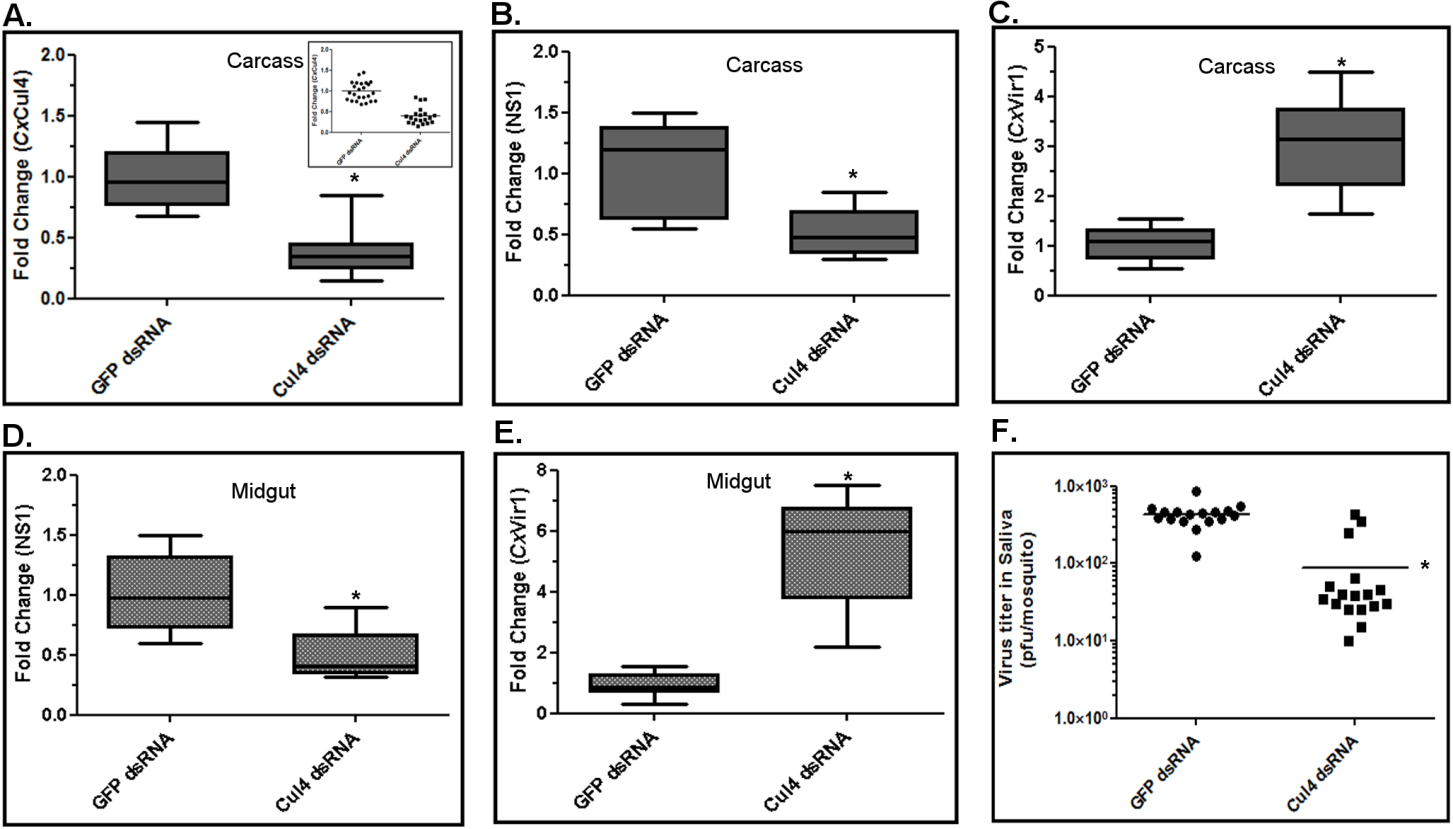
*Culex* cullin (Cul4) is pro-viral during mosquito infection. Female *Culex annulirostris* mosquitoes were microinjected with dsRNA against *Culex* Cullin (Cul4). GFP dsRNA was used as a silencing control. At 24 h post-injection, mosquitoes were blood-fed with WNV (NY99-4132 strain) for 1 h. At 12 days post-infection, mosquitoes were dissected and total RNA from the midgut and carcass was collected. **A**. Real-time RT-qPCR was performed using *Culex* Cul4 primers on mosquito carcasses. Control (GFP dsRNA) N = 24; Cul4 dsRNA N = 20. Inset shows individual mosquito data. **B**. Real-time RT-qPCR was performed using WNV NS1 primers on mosquito carcasses. **C**. Real-time RT-qPCR was performed using *Culex* Vir1 primers on mosquito carcasses. **D**. Real-time RT-qPCR was performed using *Culex* Cul4 primers on mosquito midguts. **E**. Real-time RT-qPCR was performed using WNV NS1 primers on mosquito midguts. For all real-time RT-qPCR experiments, RpL32 primers were used as an internal control. For plotting, the mean for controls was arbitrarily set at 1 (median shown) and fold-change was calculated for each sample (Student’s t-test *p < 0.005, comparing between control and Cul4-knock-down mosquitoes). **F**. Viral titer estimation conducted by plaque assay in mosquito saliva mixed with cell culture medium. Viral titers from individual saliva samples were plotted on the graph (Student’s t-test *p < 0.005, comparing between control and Cul4-knock-down mosquitoes). Control (GFP dsRNA) N = 17; Cul4 dsRNA N = 17.

To validate the significance of *Cx*Cul4 during viral infection of mosquitoes, female *Culex annulirostris* mosquitoes were microinjected with dsRNA against *Cx*Cul4. As a control, mosquitoes were microinjected with dsRNA against GFP. The mosquitoes were infected with WNV (NY99 strain) by blood-feeding 24 h post-microinjection. At 10 days post-infection, saliva from individual mosquitoes was collected in a capillary tube (see methods) and total RNA was collected from whole mosquito carcass and midgut. As a parallel experiment, mosquitoes were collected at day 2 and day 10 post-infection. Western blot was performed on whole mosquito using anti-*Cx*STAT and anti-actin antibodies. Results showed increased levels of *Cx*STAT by day 2 post-infection. The levels decreased to baseline by day 10 post-infection in control mosquitoes (GFP dsRNA); however in mosquitoes with *Cx*Cul4 dsRNA, STAT levels remained high **(Fig 5E)**. Real-time RT-qPCR results showed efficient knock-down (>60%) of *Cx*Cul4 mRNA in carcass after Cul4 dsRNA microinjections (**Fig 6A**). The results also showed a decrease (>50%) in viral RNA (NS1) in whole mosquito carcass (**Fig 6B**) and midgut (**Fig 6D**) in *Cx*Cul4 dsRNA-injected mosquitoes compared with the control, confirming pro-viral effect of *Cx*Cul4. Interestingly, real-time RT-qPCR results also showed a significant increase in *Cx*Vir1 mRNA expression in carcass (3-fold) (**Fig 6C**) and midgut (5-fold) (**Fig 6E**) in *Cx*Cul4-knockdown mosquitoes, indicating increased Jak-STAT signaling. Plaque assays performed on mosquito saliva showed a significantly lower virus titer in mosquito saliva microinjected with *Cx*Cul4 dsRNA (88 pfu/mosquito) compared with control (GFP dsRNA) (429 pfu/mosquito) (**Fig 6F**), indicating a lower likelihood of virus transmission by *Cx*Cul4 silenced mosquitoes. It should be noted that saliva from 3 mosquitoes with *Cx*Cul4 dsRNA injection showed higher viral titers (similar to controls). Further analysis showed that *Cx*Cul4 was not efficiently knocked down in these three mosquitoes (**Fig 6A, inset**). These results further validate that *Cx*Cul4 has a pro-viral effect by inhibiting Jak-STAT pathway during WNV infection in mosquitoes. It should also be noted that ~20–30% of mosquitoes from each group did not show any virus in the saliva, possibly indicative of vector competence of these mosquito species.

## Discussion

A number of studies have also been performed to identify differentially regulated genes during flavivirus infection in mammalian cells [31,36–39]. Transcriptome studies using genome-wide microarrays or next-generation deep sequencing of the transcriptome, as well as genome-wide siRNA studies, have revealed a number of pathways to be important during infection process [25,31]. These include immune pathways, apoptotic pathways, cellular transport proteins, proteasomal pathways among others.

Previous studies using microarray analyses to characterize the transcriptional response of mosquitoes to flavivirus infection have shown that a multitude of pathways appear to be involved, including the immune, cell-cycle/apoptosis, metabolic and proteasomal pathways as well as other cellular processes such as the transport machinery [10,11]. Here we adopted an unbiased approach using high-throughput deep sequencing of the mosquito cell transcriptome during a synchronized (high multiplicity) infection with WNV. Our data also identified differentially regulated genes from a number of cellular pathways of which we selected ubiquitin-proteasomal pathway for further functional analysis.

Proteasomal inhibition has been shown previously to affect replication of a wide range of viruses in mammalian cells, including herpesviruses [40], poxviruses [41], hepadnaviruses [42], adenoviruses [43], influenza viruses [44], retroviruses [45], coronaviruses [46], paramyxoviruses [47] and rotaviruses [48]. Microarray studies have also shown that several ubiquitin-related genes are induced following infection in mosquito cells [32]. The ubiquitin-proteasomal pathway is one of the major pathways involved in post-translational modifications, leading to degradation of proteins. It has also been implicated in various stages of viral infection in mammalian cells including virus entry, replication and exit [49]. The largest family of ubiquitin ligases (E3) is a group of proteins that contain a RING domain, a structural motif comprising eight cysteine and histidine residues that form the interface with E2 (ubiquitin conjugating) enzyme [18]. Substrate specificity is determined by variant RING ubiquitin ligases and also by multiprotein complexes that contain a conserved RING protein. Cullin RING ubiquitin ligases are one such family of proteins, containing eight mammalian members, each with different substrate specificity [22]. These proteins have been implicated in many cellular processes including cell-cycle regulation and cell signaling. Because of this, cullin RING ubiquitin ligases are frequently targeted by viruses to evade host immunity [33]. Viruses redirect these ligases to select specific host proteins for degradation in order to prevent the host response and promote viral replication and dissemination. For example, viruses have been shown to use cullins to prevent cellular apoptosis by degrading p53 [50] or inhibit the innate antiviral response by blocking interferon signaling [30]. In mammals, interferon is a key component of a major innate defensive response against invading viruses by activating Jak-STAT pathway and the expression of antiviral genes in neighbouring cells [51]. Some members of the *Paramyxoviridae* have been shown to hijack cullin4A ubiquitin ligase complexes to overcome the interferon response, by promoting degradation of STAT proteins. Specifically, the V protein of simian virus 5 causes degradation of STAT1 [29], while the parainfluenza virus V protein causes degradation of STAT2 via the Cul4A substrate adaptor DDB1 [52].

Recently, *Culex* Vago, which is induced in mosquitoes in response to WNV infection, was found to be functionally similar to mammalian interferon in that it is activated via the TRAF-NF-κB pathway and, upon secretion, activates the Jak-STAT pathway and an antiviral response in neighbouring cells [7,35]. Our results here suggest that *Culex* STAT is regulated by *Culex* Cul4 (mammalian Cul4A/B ortholog), which has proviral activity during WNV infection, and that WNV may target STAT for degradation by inducing *Culex* Cul4 via NS1 and NS5 proteins. Suppression of the mammalian interferon-mediated Jak-STAT pathway has been shown to be a common property of vector-borne flaviviruses. Dengue NS5 protein has been shown to target STAT2 for degradation [53] and WNV NS5 suppressed phosphorylation of STAT1 [54], leading to decreased signaling in mammalian system. Interestingly, our results suggest that the mosquito Jak-STAT pathway is also targeted by WNV via cullin RING ubiquitin ligase. Although, we have identified WNV NS1 and NS5 proteins to be responsible for upregulation of Cul4, the exact mechanism of this activation remains unknown. WNV NS1 and NS5 proteins have significantly different protein sequences, however both are involved in formation of viral replication complex. Studies are currently underway to determine whether this plays any part in the described mechanism. Our *in vivo* results also suggest that *Cx*Cul4 proviral activity may be mainly localized in the mosquito midgut and knockdown of *Cx*Cul4 increases Jak-STAT signaling in the midgut, thus decreasing the overall viral load in the body and, in turn, the saliva of these mosquitoes.

Our unbiased transcriptome analysis also indicated that other genes involved in the ubiquitin-proteasomal pathway, including E1, UBC and other E3 ligases, as well as ubiquitin-specific proteases are differentially expressed during WNV infection, suggesting that this pathway has a critical role in the invertebrate response to infection and/or the viral host evasion strategy. Our results suggest that proteasomal pathway (and *Cx*Cullin4) does not play any role in WNV entry in mosquito cells (**S5 Fig**). This is different than previously reported mechanism in mammalian cells [25]. This may be due to different mechanisms of viral entry in mammalian cells versus mosquito cells. It is also interesting that a number of reads from our data did not map to the published *Culex quinquefasciatus* transcriptome. Many of these transcripts may be expressed from unannotated genes which contribute to novel host-pathogen interactions in invertebrates.

Here we show that cullin RING ubiquitin ligase (*Cx*Cul4) plays an important role during WNV infection of *Culex* mosquitoes and dengue infection in *Aedes albopictus* cells (**S7 Fig**). *In vitro* and *in vivo* experiments show that *Culex* Cul4 (mammalian Cul4A/B ortholog) is induced by WNV infection and acts as a proviral factor by ubiquitylation of STAT in *Culex* mosquitoes, thus inhibiting the antiviral response. It remains to be seen whether mammalian Cul4A/B act in a similar manner during viral infection. This study opens up a new avenue of research in viral evasion of the mosquito immune system.

## Materials and Methods

### Cell culture and virus propagation

Hsu (*Culex quinquefasciatus*) and RML12 (*Aedes albopictus*) cells were maintained at 28°C in Leibovitz's L-15 medium (Gibco #11415) containing 10% tryptose phosphate broth solution, 15% heat-inactivated fetal bovine serum, and 1% penicillin-streptomycin solution. West Nile virus (NY99–4132 strain) was used for the study. C6/36 (*Aedes albopictus*) cells were maintained in RPMI medium at 28°C and were used to propagate the virus. Vero cells maintained in EMEM at 37°C were used for plaque assays.

### RNA preparation and next-generation sequencing

Total RNA was collected from Hsu cells (control and WNV infected) using Qiagen RNeasy kit following the manufacturer’s instructions. RNA was quantified and checked for quality using a bioanalyser. The experiment was conducted in duplicate with two RNA-Seq libraries generated for WNV-infected cells and two for uninfected control cells. Sequencing was conducted in a single lane of an Illumina HiSeq2000 (Micromon Facility, Monash University, Australia), generating more than 80 million reads per sample **(S1 Table)**.

### Sequencing analysis

Sequence reads (paired, 100 bp) were mapped using Tophat v2.0.8 (http://ccb.jhu.edu/software/tophat/index.shtml) [55] to *Culex quinquefasciatus* (Johannesburg strain, CpipJ2.1) transcripts available from VectorBase (http://www.vectorbase.org) [56]. Differential isoform expression analysis was conducted using Cufflinks v1.3.0 (http://cole-trapnell-lab.github.io/cufflinks/) [57]. Analysis of RNA sequencing quality was performed with FastQC version 0.10.1 (http://www.bioinformatics.babraham.ac.uk/projects/fastqc/) using the default options. Pathway analysis of differentially regulated transcripts was performed using Ingenuity Pathway Analysis v9.0 (Ingenuity Systems, www.ingenuity.com).

### RNA extraction and real-time RT-qPCR

Total RNA was extracted from cells using the Qiagen RNA extraction kit according to the manufacturer's protocol. Reverse transcription was performed with random hexamer primers using the First Strand Synthesis kit (Invitrogen). Real-time RT-qPCR was performed using gene-specific primers. As an internal control, real-time RT-qPCR was also performed using the housekeeping gene, RpL32. The control was set arbitrarily at 1 and fold-increase over control was calculated by the ΔΔCt method. The experiments were conducted at least three times, each in triplicates. The results were plotted in graph format as mean ± SD.

### Proteasomal/ ubiquitylation inhibitors

MG132 and PYR-41 (Sigma-Aldrich) were used at the described concentrations dissolved in DMSO. For controls, cells were treated with DMSO. For pre-treatment, cells were initially treated with MG132, followed by WNV infection for one hour. This was followed by replacing the medium with medium containing MG132 at appropriate concentrations.

### Cell lysis and western blotting

Cells were lysed in RIPA lysis buffer (25 mM Tri-HCl, 150 mM NaCl, 1% NP-40, 0.1% SDS) containing protease inhibitor (Halt protease inhibitor cocktail, Pierce). The cell lysates were collected by centrifugation at 16,000 × *g* for 10 min at 4°C. Protein samples (10 μg) were loaded onto polyacrylamide gradient gels (4–12%). After electrophoresis and transfer to nitrocellulose membranes, proteins were blotted using anti-*Culex* STAT (rabbit polyclonal against peptide VVIVHGNQEPQSWATITWDNAFADINRV PFHVPDKVSWNLLAEALNTKYRASTGRSMTQENMHFLC), anti-WNV NS1, anti-WNV NS5 [58] or anti-V5 (Invitrogen) antibody followed by anti-rabbit or anti-mouse secondary antibodies. After adding substrate, the membrane was exposed to film to detect protein levels. Anti-β-actin antibody (Abcam) was used in immunoblots as a loading control.

### Plaque assays

Plaque assays were performed as previously described [7]. In brief, supernatant media from cells infected with WNV (10-fold dilutions) were added onto confluent Vero cell monolayers in 6-well plates. After 1 h incubation at 37°C, the cells were overlaid with medium containing agar. Plaques formed within 72 hpi were counted and the results were plotted graphically. The experiments were conducted at least twice, each with duplicates.

### dsRNA preparation and transfection

Gene-specific dsRNA (~400 nt) were prepared using the MEGAscript RNAi kit according to the manufacturer's protocol. dsRNAs were transfected into Hsu cells using Cellfectin according to a previously described protocol [7]. dsRNA against green fluorescent protein (GFP) was used as a knock-down specificity control.

### Luciferase assay activity

STAT activation experiments were performed as previously described [59]. The STAT reporter plasmids were kindly provided by Prof. Martin Zeidler (University of Sheffield) [60]. In brief, Hsu cells were transfected with STAT reporter plasmid p6x2DRAF-Luc (multimerised Drosophila STAT-responsive element with a *Firefly* luciferase reporter) and a control plasmid, pAct-*Renilla* (*Renilla* luciferase gene under control of the Drosophila actin 5C promoter for constitutive expression). Cells were also transfected with dsRNA against Cul4 or GFP. Cells were stimulated with heat inactivated *E*. *coli*, WNV (MOI = 1) or PBS (Control) for 1h. Luciferase activity was measured at 16 h post-stimulation. Heat-inactivated bacteria were prepared by incubating 1μl of *E*. *coli* DH5α in 5 ml LB medium at 37°C for 16 h. Cells were harvested by centrifugation and resuspended in 0.5 ml of PBS. Heat inactivation was achieved by incubating the cells for 10 min at 90°C. 1 μl of the suspension was used per well.

### Ubiquitylation determination

Hsu cells under various conditions were treated with MG132 (1 μM) for 6 hours to prevent degradation of ubiquitylated proteins. Immunoprecipitation was performed using Pierce Crosslink IP kit (Thermo Scientific) following manufacturer’s protocol. Briefly, cells were lysed using lysis buffer and after preclearing were incubated with anti-ubiquitin antibody (Abcam) crosslinked to beads in column for 18 hours. Flow-through was collected and immunoprecipitate was eluted using elution buffer. The samples were separated on polyacrylamide gel and Western blot was performed using anti-ubiquitin and anti-*Cx*STAT antibodies.

### Mosquito maintenance and viral infections

*Culex annulirostris* mosquitoes were maintained in a diurnal cycle (12h/12h) with temperatures alternating for 23 and26°C and 65% humidity. Three to five day-old female mosquitoes (n = 40) were blood-fed on chicken skin membranes with WNV (1.13×10∧6 pfu/ml) or 199 medium (as control) and the mosquitoes were incubated at 25°C and 65% humidity in an environmental cabinet (Thermoline Scientific, Smithfield, Australia) with a wet cotton pad (10% sucrose solution) provided daily as a food source. At 24 hpi, surviving females were collected for analysis. For this, mosquito midguts were dissected and homogenized using a bead-beater. RNA extracted using the RNeasy kit (Qiagen) by pooling four samples and was used for real-time RT-qPCR as described above.

### Mosquito microinjection and saliva collection

Three to five day-old female mosquitoes (n = 40) were microinjected with dsRNA against *Cx*Cul4 or GFP (200 ng/mosquito). Mosquitoes were blood-fed with WNV (1.13×10∧6 pfu/ml) one day later and were maintained in a diurnal cycle (23/26°C) in an environmental cabinet. At 10–12 days post-infection, saliva was collected in capillary tubes containing 5 μl FCS for 10 min using a protocol described previously [61]. Midguts were dissected and homogenized using a bead-beater. RNA was extracted using the RNeasy kit from the midgut and carcass of individual mosquitoes and was used for real-time RTqPCR as described above. Saliva from each mosquito was diluted in 300 μl of L-15 medium and was used to determine viral titer by plaque assay as described above.

### Statistical analysis

Standard error of the mean (SEM) was calculated and data analyzed using the non-paired Student's *t*-test for single mean comparisons.

## Supporting Information

S1 Table Mapping summary of the transcriptome sequencing. (DOCX)

S2 Table List of transcripts differentially regulated after WNV infection. (XLS)

S1 Fig Cell viability assay for MG132 and PYR41. (DOCX)

S2 Fig Contribution of extracellular viral RNA for real time RT-qPCR. (DOCX)

S3 Fig Protein sequence alignment of human Cul4A, human Cul4B and *Culex* Cullin. (DOCX)

S4 Fig Silencing efficiency for CxCul4 using dsRNA. (DOCX)

S5 Fig Involvement of CxCul4 during viral internalization. (DOCX)

S6 Fig Western blot on Hsu cell lysates overexpressing WNV genes, using anti-V5 antibody. (DOCX)

S7 Fig Real time RT-qPCR using Aedes Cullin4-specific primers after Dengue virus (DENV) infection of Aedes albopictus cells. (DOCX)
