# Supplementary material for: Cullin4 Is Pro-Viral during West Nile Virus Infection of Culex Mosquitoes
Source: PLoS Pathog. 2015 Sep 1;11(9):e1005143. doi: 10.1371/journal.ppat.1005143 (PMC4556628; doi:10.1371/journal.ppat.1005143)
Supplement: S1 Table — (DOCX) [file ppat.1005143.s001.docx]

Supplementary Table 1.

Mapping summary

| **Sample** | **Replicate** | **No. Transcripts^1^** | **Pearson Coefficient^2^** |
| --- | --- | --- | --- |
| Control | C#1 | 13455 | 0.994 |
|  | C#2 | 13238 |  |
| West Nile virus infection | W#1 | 14231 | 0.991 |
|  | W#2 | 14734 |  |

^1^ Number of the annotated transcripts in *Culex quinquefasciatus* (Vectorbase) assembled with the sequence reads

^2^ Pearson correlation coefficient calculated for replicate
