# Supplementary material for: Cullin4 Is Pro-Viral during West Nile Virus Infection of Culex Mosquitoes
Source: PLoS Pathog. 2015 Sep 1;11(9):e1005143. doi: 10.1371/journal.ppat.1005143 (PMC4556628; doi:10.1371/journal.ppat.1005143)
Supplement: S1 Fig — (DOCX) [file ppat.1005143.s003.docx]

**Supplementary Figure 1.**

Hsu cells were treated with increasing concentrations of MG132 or PYR41 (0, 0.1, 1, 10, 25 uM) for 48 hours. Cells were collected in PBS, stained for trypan blue and counted using hemocytometer. The results are plotted here as percent survival compared with cells treated with 0 uM MG132/PYR41 but with equivalent amount of DMSO (diluent). Error bars represents standard error from three separate experiments with assays performed in triplicate (Student’s t-test *p < 0.05, comparing between control (0) and MG132/Pyr41-treated cells).


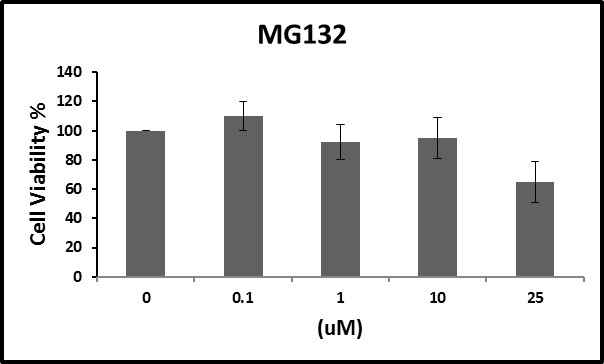


*


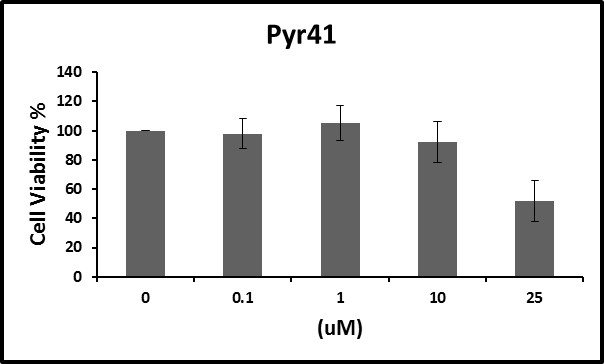


*
