# Supplementary material for: Cullin4 Is Pro-Viral during West Nile Virus Infection of Culex Mosquitoes
Source: PLoS Pathog. 2015 Sep 1;11(9):e1005143. doi: 10.1371/journal.ppat.1005143 (PMC4556628; doi:10.1371/journal.ppat.1005143)
Supplement: S2 Fig — (DOCX) [file ppat.1005143.s004.docx]

**Supplementary Figure 2.**

Hsu cells were infected with WNV (MOI 10) at 4C for 30 mins and then either left at 4C or incubated at 30C. Some cells were pre-treated with MG132 (10 uM) before infecting with WNV. Real time RT-qPCR using NS1 primers conducted on total RNA from cells collected at 6 hpi showed no (or minimal contribution from extracellular viral RNA).
