# Supplementary material for: Cullin4 Is Pro-Viral during West Nile Virus Infection of Culex Mosquitoes
Source: PLoS Pathog. 2015 Sep 1;11(9):e1005143. doi: 10.1371/journal.ppat.1005143 (PMC4556628; doi:10.1371/journal.ppat.1005143)
Supplement: S3 Fig — (DOCX) [file ppat.1005143.s005.docx]

Suppl. Fig 3 Protein sequence alignment of human Cul4A, human Cul4B and *Culex* Cullin

hCul4A --MADEAPRKGSFS----------------------ALVGRTNGLTKPAALAAAPAKPGG 36

hCUL4B --MAEESSSSSSSSSPTAATSQQQQLKNKSILISSVASVHHANGLAKSSTTVSSFANSK- 57

CxCullin MNLAENDRKRANFS-----------------------ALSNTNGAVIKMTTNTGTGKPG- 36

:*:: .. * : .:** . : :. .:.

hCul4A AGGSKKLVIKNFRDRPRLPDNYTQDTWRKLHEAVRAVQSSTSIRYNLEELYQAVENLCSH 96

hCUL4B PGSAKNLVIKNFKDKPKLPENYTDETWQKLKEAVEAIQNSTSIKYNLEELYQAVENLCSY 117

CxCullin --DIKKIVIKNFKTKPTLPENYQETTWQKLREAVIAIQLSKRIEYSLEELYQAVENMCSH 94

. *::*****: :* **:** : **:**:*** *:* *. *.*.**********:**:

hCul4A KVSPMLYKQLRQACEDHVQAQILPFREDSLDSVLFLKKINTCWQDHCRQMIMIRSIFLFL 156

hCUL4B KISANLYKQLRQICEDHMKAQIHQFREDSLDSVLFLKKIDRCWQNHCRQMIMIRSIFLFL 177

CxCullin KMDSQLYVNLTALAEQHVKANITPFMAESIDKLVYLKKMNDCWQSHCQQMIMIRSIFLYL 154

*:.. ** :* .*:*::*:* * :*:*.:::***:: ***.**:**********:*

hCul4A DRTYVLQNSTLPSIWDMGLELFRTHIISDKMVQSKTIDGILLLIERERSGEAVDRSLLRS 216

hCUL4B DRTYVLQNSMLPSIWDMGLELFRAHIISDQKVQNKTIDGILLLIERERNGEAIDRSLLRS 237

CxCullin DRTYVLQNPTVHSIWDMGLELFRDHIAMNTLVQARTVEGILILIEKERNGDAVDRALLKS 214

********. : *********** ** : ** :*::***:***:**.*:*:**:**:*

hCul4A LLGMLSDLQVYKDSFELKFLEETNCLYAAEGQRLMQEREVPEYLNHVSKRLEEEGDRVIT 276

hCUL4B LLSMLSDLQIYQDSFEQRFLEETNRLYAAEGQKLMQEREVPEYLHHVNKRLEEEADRLIT 297

CxCullin LLRMLSDLQIYKEAFEQKFLVATKHLYQSEGQAKMEVLEVPEYLLHVDKRLQEENERLLH 274

** ******:*:::** :** *: ** :*** *: ****** **.***:** :*::

hCul4A YLDHSTQKPLIACVEKQLLGEHLTAILQKGLDHLLDENRVPDLAQMYQLFSRVRGGQQAL 336

hCUL4B YLDQTTQKSLIATVEKQLLGEHLTAILQKGLNNLLDENRIQDLSLLYQLFSRVRGGVQVL 357

CxCullin YLDSCTKHQLIVTVERQLITEHITGILQKGLDQLLEENRLTDLTLLYSLFSRVKNGTIEL 334

*** *:: **. **:**: **:*.******::**:***: **: :*.*****:.* *

hCul4A LQHWSEYIKTFGTAIVINPEKDKDMVQDLLDFKDKVDHVIEVCFQKNERFVNLMKESFET 396

hCUL4B LQQWIEYIKAFGSTIVINPEKDKTMVQELLDFKDKVDHIIDICFLKNEKFINAMKEAFET 417

CxCullin CASFNAYIKKKGRTIVIDPEKDKSMVQDLLDFKDKLDNIVTKCFDKNEKFSNSLREAFEF 394

: *** * :***:***** ***:*******:*::: ** ***:* * ::*:**

hCul4A FINKRPNKPAELIAKHVDSKLRAGNKEATDEELERTLDKIMILFRFIHGKDVFEAFYKKD 456

hCUL4B FINKRPNKPAELIAKYVDSKLRAGNKEATDEELEKMLDKIMIIFRFIYGKDVFEAFYKKD 477

CxCullin FVNQRSNKPAELIAKYVDMKLRAGNKEATEEELEQILDKIMVQFRFIHGKDVFEAFYKKD 454

*:*:*.*********:** **********:****: *****: ****:************

hCul4A LAKRLLVGKSASVDAEKSMLSKLKHECGAAFTSKLEGMFKDMELSKDIMVHFKQ--HMQN 514

hCUL4B LAKRLLVGKSASVDAEKSMLSKLKHECGAAFTSKLEGMFKDMELSKDIMIQFKQVKYMQN 537

CxCullin LAKRLLVGKSASVDAEKSMLSKLKQECGGGFTSKLEGMFKDMELSRDINIAFRQYMANSE 514

************************:***..***************:** : *:* .:

hCul4A QSDSGPIDLTVNILTMGYWPTYTPMEVHLTPEMIKLQEVFKAFYLGKHSGRKLQWQTTLG 574

hCUL4B QNVPGNIELTVNILTMGYWPTYVPMEVHLPPEMVKLQEIFKTFYLGKHSGRKLQWQSTLG 597

CxCullin GKELQNIDLTVNILTMGFWPTYPVMEVTLPQELLQYQSIFNKFYLAKHSGRKLQWQPTLG 574

. *:*********:**** *** *. *::: *.:*: ***.**********.***

hCul4A HAVLKAEFKEGKKEFQVSLFQTLVLLMFNEGDGFSFEEIKMATGIEDSELRRTLQSLACG 634

hCUL4B HCVLKAEFKEGKKELQVSLFQTLVLLMFNEGEEFSLEEIKQATGIEDGELRRTLQSLACG 657

CxCullin HCVLKARFDAGPKDLQVSLFQALVLLLFNYSPTITFEEIKAAINIEDGELRRTLQSLACG 634

*.****.*. * *::******:****:** . :::**** * .***.************

hCul4A KARVLIKSPKGKEVEDGDKFIFNGEFKHKLFRIKINQIQMKETVEEQVSTTERVFQDRQY 694

hCUL4B KARVLAKNPKGKDIEDGDKFICNDDFKHKLFRIKINQIQMKETVEEQASTTERVFQDRQY 717

CxCullin KARVVSKIPKGREVEDNDKFQFNNEFTNKLFRIKINQIQMKETTEEQKATEERVYQDRQY 694

****: * ***:::**.*** *.:*.:***************.*** :* ***:*****

hCul4A QIDAAIVRIMKMRKTLGHNLLVSELYNQLKFPVKPGDLKKRIESLIDRDYMERDKDNPNQ 754

hCUL4B QIDAAIVRIMKMRKTLSHNLLVSEVYNQLKFPVKPADLKKRIESLIDRDYMERDKENPNQ 777

CxCullin QIDAAIVRIMKMRKTLSHNLLISELYKQLTFPVKPADLKKRIESLIDRDYMERDKDNQNQ 754

****************.****:**:*:**.*****.*******************:* **

hCul4A YHYVA 759

hCUL4B YNYIA 782

CxCullin YNYVA 759

*:*:*
