# Supplementary material for: Cullin4 Is Pro-Viral during West Nile Virus Infection of Culex Mosquitoes
Source: PLoS Pathog. 2015 Sep 1;11(9):e1005143. doi: 10.1371/journal.ppat.1005143 (PMC4556628; doi:10.1371/journal.ppat.1005143)

**Supplementary Figure 4.**

Hsu cells were transfected with dsRNA against CxCul4 (Cul4 dsRNA), followed by infection with WNV for 48 hours. As a control, cells were either not transfected with dsRNA (No dsRNA) or transfected with dsRNA against GFP (GFP dsRNA). Real time RT-PCR was conducted on total RNA from cells using CxCul4 primers to assess efficiency of silencing.


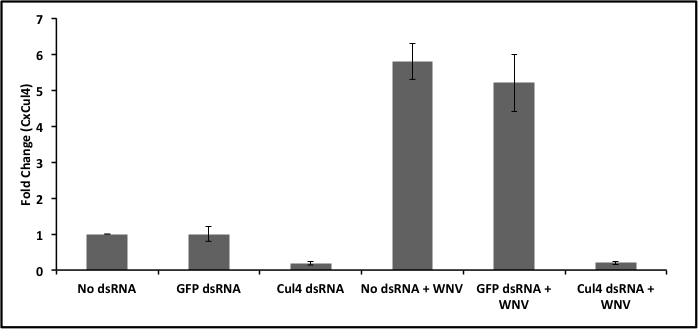

Supplement: S4 Fig — (DOCX) [file ppat.1005143.s006.docx]
