# Supplementary material for: Cullin4 Is Pro-Viral during West Nile Virus Infection of Culex Mosquitoes
Source: PLoS Pathog. 2015 Sep 1;11(9):e1005143. doi: 10.1371/journal.ppat.1005143 (PMC4556628; doi:10.1371/journal.ppat.1005143)

**Supplementary Figure 5.**

Hsu cells were transfected with plasmid overexpressing CxCul4 (CxCul4), followed by infection with WNV at 4C for 30 mins. As a control, cells were transfected with empty vector (Control). Cells were then incubated at 30C for 6 hours and real time RT-PCR was conducted on total RNA from cells using WNV-NS1 primers to assess internalization of virus.


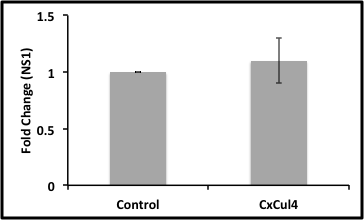

Supplement: S5 Fig — (DOCX) [file ppat.1005143.s007.docx]
