# Supplementary material for: Cullin4 Is Pro-Viral during West Nile Virus Infection of Culex Mosquitoes
Source: PLoS Pathog. 2015 Sep 1;11(9):e1005143. doi: 10.1371/journal.ppat.1005143 (PMC4556628; doi:10.1371/journal.ppat.1005143)

Supplementary Figure 6. Western blot on Hsu cell lysates overexpressing WNV genes, using anti-V5 and anti-NS1 antibodies.


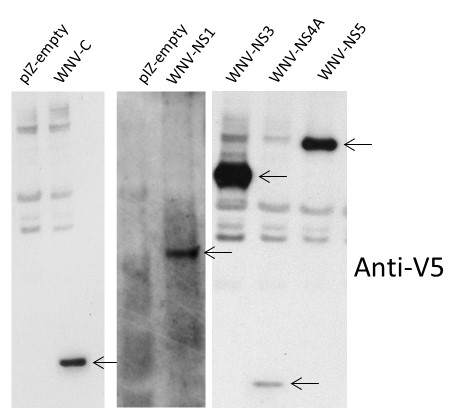

Supplement: S6 Fig — (DOCX) [file ppat.1005143.s008.docx]
