# Supplementary material for: Cullin4 Is Pro-Viral during West Nile Virus Infection of Culex Mosquitoes
Source: PLoS Pathog. 2015 Sep 1;11(9):e1005143. doi: 10.1371/journal.ppat.1005143 (PMC4556628; doi:10.1371/journal.ppat.1005143)

**Supplementary Figure 7.**

RML12 cells (*Aedes albopictus*) were infected with DENV for 48 hours. Total RNA as collected and real time RT-qPCR was performed using Aedes Cul4 specific primers. Control represents mock-infected cells. RpL32 primers were used as an internal control. Error bars represent standard error from three separate experiments with assays performed in triplicate (Student’s t-test *p < 0.05, comparing between mock and DENV infected cells).


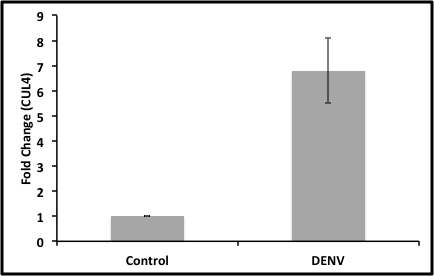

Supplement: S7 Fig — (DOCX) [file ppat.1005143.s009.docx]
